# Synthesis of New Hydrogels Involving Acrylic Acid and Acrylamide Grafted Agar-Agar and Their Application in the Removal of Cationic Dyes from Wastewater

**DOI:** 10.3390/gels9060499

**Published:** 2023-06-19

**Authors:** Amina Betraoui, Nesrinne Seddiki, Rafika Souag, Nabila Guerfi, Abdelhabib Semlali, Taieb Aouak, Djamel Aliouche

**Affiliations:** 1 Laboratory of Polymers Treatment and Forming, F.S.I., M’Hamed Bougara University, Boumerdes 35000, Algeria; 2 Research Unit, Materials, Processes and Environment (URMPE), University of Boumerdes, Boumerdes 35000, Algeria; 3 Centre de Recherche en Technologiedes Semi-Conducteurs pour l’Energétique (CRTSE), 02 Bd Frantz Fanon BP140, 7 Merveilles, Algiers 16038, Algeria; 4 Groupe de Recherche en Écologie Buccale, Faculté de Médecine Dentaire, Université Laval, Quebec City, QC G1V 0A6, Canada; 5 Chemistry Department, College of Science, King Saud University, P.O. Box 2455, Riyadh 11451, Saudi Arabia

**Keywords:** polysaccharide Agar-Agar, polyacrylamide-grafted-Agar, polyacrylic acid-grafted-Agar, swelling properties, water adsorption, removing cation dye, methylene blue

## Abstract

Polyacrylic Acid grafted Agar-agar (AAc-*graf*-Agar), and polyacrylamide grafted Agar-Agar (AAm-*graf*-Agar) have been synthesised by free radical polymerisation route initiated by ammonium peroxodisulphate (APS), the grafted polymers were characterised by FTIR, TGA and SEM methods. The swelling properties were studied in deionised water and saline solution at room temperature. The prepared hydrogels were examined by removing cationic methylene blue (MB) dye from the aqueous solution, in which the adsorption kinetics and isotherms models were also investigated. It was found that the pseudo-second-order and Langmuir equations are the most suitable for the different sorption processes. The maximum dye adsorption capacity was 1035.96 mg∙g^−1^ for AAc-*graf*-Agar in pH medium 12 and 1015.7 mg∙g^−1^ for AAm-*graf*-Agar in neutral pH medium. This indicates that the AAc-*graf*-Agar hydrogel could be an excellent adsorbent for removing MB from aqueous solutions.

## 1. Introduction

One of the most significant categories of pollutants is dyes [1]. Dye pollution comes from the textile and printing sectors and poses a number of risks to the aquatic environment and public health. However, many textile dyes are recognised to be highly toxic, mutagenic, and carcinogenic substances [2]. Therefore, the elimination of synthetic dyes is crucial. The removal of dyes from water can be performed using a variety of methods, including chemical oxidation, ozonation, membrane filtering, flotation, and adsorption.

**Scheme 1:**
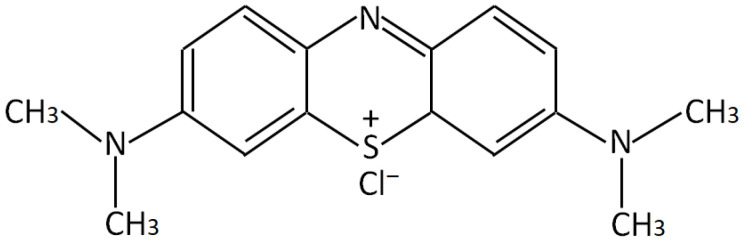
Chemical structure of methylene blue (MB).

Due to its operational simplicity and high efficiency, adsorption is regarded as one of the most performing method [3]. 3,7-bis(dimethylamino)-phenazathionium chloride or methylene blue (MB) (Scheme 1) is a cationic dye that is considered one of the oldest synthetic dyes belonging to the thiazine family [4,5].

MB is also considered one of the best options for colouring fabrics, particularly wool, silk, and other natural fibres, due to its vibrant blue hue, excellent water solubility and affordability. This dye is also considered a potential medication for the treatment of encephalopathy, chronic Lyme disease, vasoplegic syndrome, and cyanide poisoning [6]. It has a long history of use in the treatment of malaria. According to recent studies, MB dye may be used to treat fatal respiratory diseases due to COVID-19. MB is also known to have anti-inflammatory, antidepressant and cardio-protective properties. Other important applications for MB include staining, as an indicator, as in the case of the photosensitiser [7]. For the removal of various dyes from aqueous solutions under various operating circumstances, researchers have used a variety of efficient and biodegradable adsorbents obtainable from natural resources. Particular attention was paid to the super absorbent hydrogel [8].

This is how Kara et al. [9] investigated the removal of cationic MB dye from wastewater using sodium periodate-modified nanocellulose (NaIO_4_-NC) prepared from Eichhornia crassipes. The adsorption process was performed using Langmuir and Freundlich isotherm models, and the result obtained revealed a maximum adsorption efficiency of 90.91 mg·g^−1^ and colour removal of 78.1% at optimum 30 mg·L^−1^, 60 min. Shkour and Nasar [10] studied the removal of MB dye using citrus limetta peel (CLP) as a low-cost adsorbent, and it was found to be best represented by Langmuir adsorption isotherm with a maximum adsorption capacity for monolayer coverage of 227.3 mg·g^−1^.

Brown clay (BC) was modified with didodecyl dimethyl ammonium bromide (DDAB) to produce a sorbent (DDAB-BC) [11]. The resulting material, when applied to the removal of MB dye from aqueous media, showed a maximum efficiency of 98%) and a maximum sorption capacity of 164 mg·g^−1^). Deacetylated cellulose acetate (DA)@polydopamine (PDA) composite nanofiber membrane fabricated by electrospinning and surface modification was used by Cheng et al. [12] as membrane as a highly efficient adsorbent for removing MB from an aqueous solution. The adsorption capacity of the DA@PDA nanofiber membrane reached up to 88.2 mg·g^−1^ at 25 °C and a pH of 6.5 after adsorption for 30 h, which represents about 8.6 times higher than that of DA nanofibers.

Recently, Mosoarca et al. [13] employed raspberry (*Rubus idaeus*) leaves converted to powder as a natural lignocellulosic low-cost adsorbent for MB dye removal from aqueous solutions, and the main result obtained was a maximum adsorption capacity of 244.6 mg·g^−1^ higher compared to other adsorbents based on plant leaves. The contact time was the factor with the highest influence on the process, while the temperature had the lowest influence.

Geopolymer and magnetite/geopolymer composite (MGP) with a specific surface area of 26.604 m^2^·g^−1^ and 69.04 m^2^·g^−1^, respectively, were also used in the MB dye removal [14]. The results obtained indicated that a mixture of Fe_3_O_4_ and geopolymer with 10% magnetite to geopolymer had high adsorption performance on MB, with a removal rate of over 95%, which was much greater than that of separate mesoporous geopolymers.

Recently, the hydroxyl groups modified on the surface metal–organic framework UiO-66 were used to improve the adsorption capacity of the cationic MB dye [15]. The authors reported that an excellent MB adsorption performance was observed in the pH medium ranging between 3 and 11, and the U-(OH)_2_ has a more stable regeneration capacity just using solvent washing. Bio-adsorbents, such as jackfruit leaf powder, were employed by Noor et al. [16] in removing MB dye ingredients from wastewater due to their high kinetics and capacity. The maximum adsorption capacity at equilibrium was found to be 271 mg·g^−1,^ and adsorption followed Langmuir isotherm. The kinetics study provided evidence of instantaneous adsorption, while the zero-point charge of the adsorbent was 3.9.

Rahman et al. [17] used rice husk-activated carbon, a basic dye, as an adsorbent for the removal of methylene blue dye from aqueous solutions. Various experimental parameters, such as adsorbent dosage and particle size, initial dye concentration, pH and flow rate, were investigated in the column process. It was found that the maximum uptakes of methylene blue by activated rice husk carbon at optimised conditions were found to be 97.15%.

Recently, a new generation of adsorbents based on the plant was also investigated by Gul et al. [18] to remove dye from wastewater. These authors used leaves of the Adiantum capillus-veneris plant to remove commonly used textile dyes from aqueous dye solutions and wastewater. Different factors that affected the performance of this method were studied, and the results obtained revealed excellent results compared to the commercial adsorbents. Indeed, the leaves of the Adiantum capillus-veneris plant revealed a maximum removal of 90.36% crystal violet dye (adsorption capacity of 9.05 mg·g^−1^) without any treatment to activate or alter the surface chemistry of the biosorbent. In another work, these same authors [19] employed Banyan (*Ficus benghalensis*, *F. benghalensis*) tree leaves to remove brilliant green (BG) cationic dye from an aqueous solution. Different parameters influencing the efficiency of these bioabsorbants were studied, and the results obtained were a maximum percent removal of 97.3% and an adsorption capacity (Qe) of 19.5 mg·g^−1^.

The sustainable removal of methyl red (MR) dye, known as a dye with adverse effects on the environment and human health, was studied by Gul et al. [20], in one of his works, uses biomass derived from the bark of the *Dodonaea viscosa* (Hopbush) plant. Hopbush bark shows efficient adsorption of MR, up to 73%, under optimal conditions in aqueous media. It was found that the elimination of MR (500 ppm) was at pH medium 1 and a contact time of 75 min. Moreover, it was revealed that the adsorption capacity of bark powder surpassed animal charcoal, silica gel and powdered flowers, as well as leaves of the same species.

Clay-organic composites have also been used as an adsorbent for dye released by textile industries in waters. This is how Ozola-Davidane et al. [21] studied the removal of the model organic contaminant, Congo red dye, from aqueous solutions using clays modified with ionic liquids based on imidazolium. The results obtained revealed that the modified clays have a much higher Congo red sorption capacity compared to unmodified bentonite, in which the maximum Congo red sorption capacity of 150 mg·g^−1^ was observed for bentonite modified with 1-dodecyl-3-methylimidazolium chloride.

Hydrogels are a class of materials that consist of a three-dimensional network of polymers that absorb large amounts of water [22]. They easily separate from the medium after the adsorption process [23]. Graft copolymers are a particular type of branched polymers that have the potential to produce desired characteristics that are not present in the parent backbone.

Grafting mainly affects the side groups of the polymer chains; as a result, the main skeleton is unaffected, and the molecular characteristics of the skeleton are only slightly altered [24]. The free radical sites on this premade polymer are fabricated outside. The main processes for prepared graft copolymers use chemical free radical initiators (conventional method), such as ammonium peroxodisulphate (APS), to produce free radical sites on the premade polymer’s backbone, where the monomer attaches, and the graft chain is created [25,26]. Natural polysaccharides are generally non-toxic, biodegradable, recyclable, economical and harmless to the environment. In order to remove colours from water, they are therefore widely used in the creation of novel adsorbent materials [3].

**Scheme 2:**
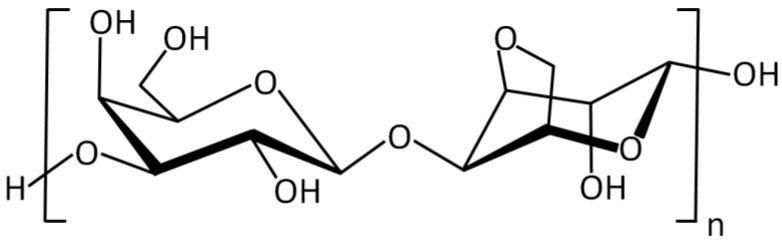
Chemical structure of Agar-Agar.

Agar-Agar is a biopolymer (Scheme 2) that is naturally occurring, biodegradable, thermo-reversible, and reasonably priced. It is frequently employed as a thickening, coagulant, emulsifier, stabiliser, gelling, and film-forming agent in industrial applications. The range of applications for agar has grown recently [27].

Agar-Agar contains galacto-pyranose dimers connected by alternating 1,3- and 1,4-linked galactose and 3,6-anhydro—galactopyranose units [28]. Agar, which is generated from specific types of red algae and used as a component in meals and microbial cultures, is another significant natural polysaccharide. Agar has been widely used in the food industry as stabilisers, thickeners, water-holding carriers, gel-forming media, and drug delivery systems.

Networks created by crosslinking natural polymers with vinyl monomers and glycine are thought to be promising area for further study. Hydrogels are crucial for widespread use in environmental applications because they have a strong adsorption affinity to remove various pollutants from aqueous solutions, even at lower concentrations [29]. The acidic carboxylic group present in the structure of a monomer unit, such as acrylic acid or methacrylic acid in a polymer, are highly desirable in order to increase the adsorption and swelling properties of hydrogels.

Acrylic polymers form complexes with metal cations and cationic dyes because they contain electron-withdrawing atoms like oxygen [30]. The hydroxyl group in these polymers also contributes to the formation of inter-chain hydrogen bonds with water molecules and also with dye molecules. The hydrogel behaviour of patients towards acrylic molecules is sensitive to the average pH because the carboxyl group has a low acidity [31].

The aim of this work is to prepare and characterise hydrogels prepared by grafting acid acrylic on the main chain of Agar (AAc-*graf*-Agar) and, on the other hand, acrylamide on the backbone of this same biopolymer (PAAm-*graf*-Agar). The structures of the hydrogels obtained are confirmed by Fourier transform infrared (FTIR). The thermal behaviour and the surface morphology of these prepared materials are examined by thermogravimetry analysis (TGA) and scanning electron microscopy (SEM), respectively. The swelling dynamics of the prepared hydrogels in deionised water and in saline solution are investigated. The removal of cationic MB dye from water is widely studied in this work, in which the effects of pH medium, temperature, contact time, dye concentration and dosage of the adsorbent on the efficiency of the adsorption process are examined. This work is completed by a kinetic study of adsorption in which the isotherms of the grafting hydrogel have also been considered.

## 2. Results and Discussion

### 2.1. Characterization

The chemical structures of the prepared hydrogels were characterised by FTIR analysis, their thermal stability by TGA and their surface morphology was examined by SEM, and the results obtained are detailed in the following sections.

#### 2.1.1. FTIR Analysis

**Figure 1:**
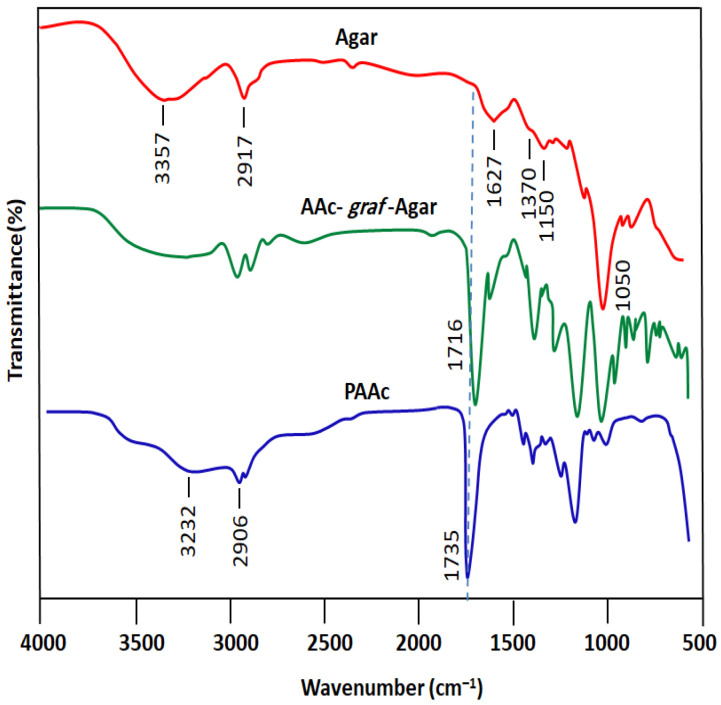
Comparison of FTIR spectra of AAc-*graf*-Agar with their pure corresponding homopolymers.

The FTIR spectra of AAc-*graf*-Agar and their corresponding pure poly (acrylic acid) (PAAc) and Agar homopolymers are grouped for comparison in Figure 1. The spectrum of the native Agar shows a broad absorption band at 3305 cm^−1^ and 3299 cm^−1^ assigned to the stretching frequency of the hydroxyl group (-OH), as well as intramolecular and intermolecular hydrogen bonds. The absorption bands, including free, inter-, and intra-molecular hydroxyl groups, ranged between 3700 and 3000 cm^−1^. The bands observed at 2935 cm^−1^ and 2933 cm^−1^ are attributed to the C–H stretching vibration of methynyl group (-CH_2_)-. The signal observed around 1643–1639 cm^−1^ is assigned to the absorbed water [32]. In the fingerprint region, the peaks localised at 1004 cm^−1^ and 987 cm^−1^ are attributed to the stretching vibrations of the C-O-C group of 3,6-anhydro-D-galactose [33]. The spectrum of native PAAc shows a band at 1117 cm^−1^ characterising the carbonyl groups of this homopolymer and weaker peaks at 1451 and 1403 cm^−1,^ which are associated with scissor and bending vibrations of -CH- and CH-CO- groups, respectively. The absorption bands observed at 1235 and 1170 cm^−1^ are attributed to the coupling between in-plane O-H bending and C-O stretching of neighbouring carboxyl groups [34,35]. As can be seen from these traces, all the absorption bands attributed to the two homo-polymers are present in the spectrum of the AAc-*graf*-Agar copolymer. The comparison of these spectra also reveals a shift in the absorption band of the carbonyl of the acrylyl unit towards the low wave number and also that of the hydroxyl function. This indicates a rearrangement of the hydrogen bonds caused by the incorporation of the agar units, also bearing the hydroxyl groups, into the poly(acrylic acid) chains.

**Figure 2:**
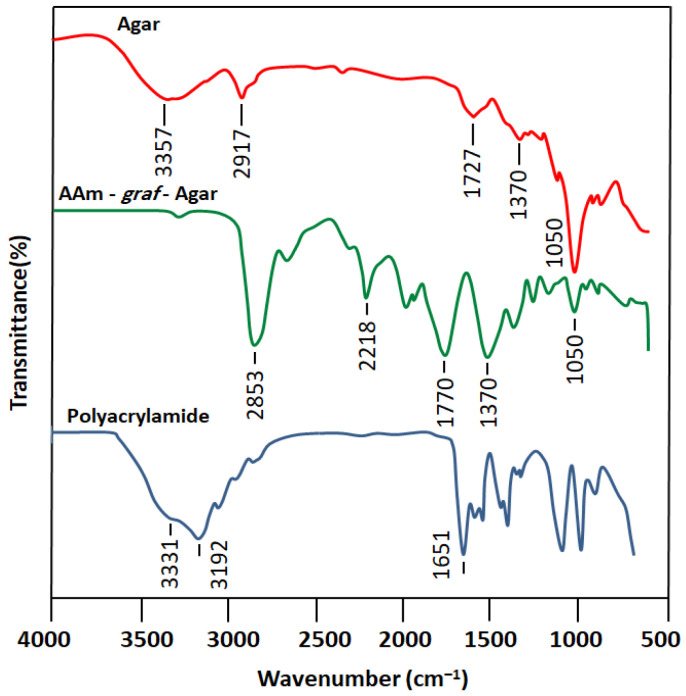
Comparative FTIR spectra of AAm-*graf*-Agar hydrogel and their pure components.

For the native polyacrylamide (PAAm) shown in Figure 2, this polymer shows their characteristic absorption bands at 3420 cm^−1^ attributed to the NH stretch, 2924 cm^−1^ assigned to the symmetric –CH_2_- stretch in the polymer chain backbone and that at 1680 cm^−1^ attributed to the carbonyl of the acrylamidyl monomer unit.

The comparison of the FTIR spectrum of the AAm-*graf*-Agar copolymer with those of its pure components, as shown in Figure 2, as in the case of the AAc-*graf*-Agar copolymer, reveals the presence of all the absorption bands specific to both homopolymers. This same trace also reveals a shift to the right of the signals attributed to the hydroxyl and carbonyl groups belonging to the two different units of the copolymer in addition to a broad band of the carbonyl of the acrylamidyl unit. According to the literature [36,37], an important shift to the right or a broadening of the absorption bands of these functions testifies to the presence of hydrogen bonds. This also reveals an important rearrangement of the hydrogen bonds caused by the introduction of the agar units in the chains of the copolymer.

#### 2.1.2. TG-Analysis

**Figure 3:**
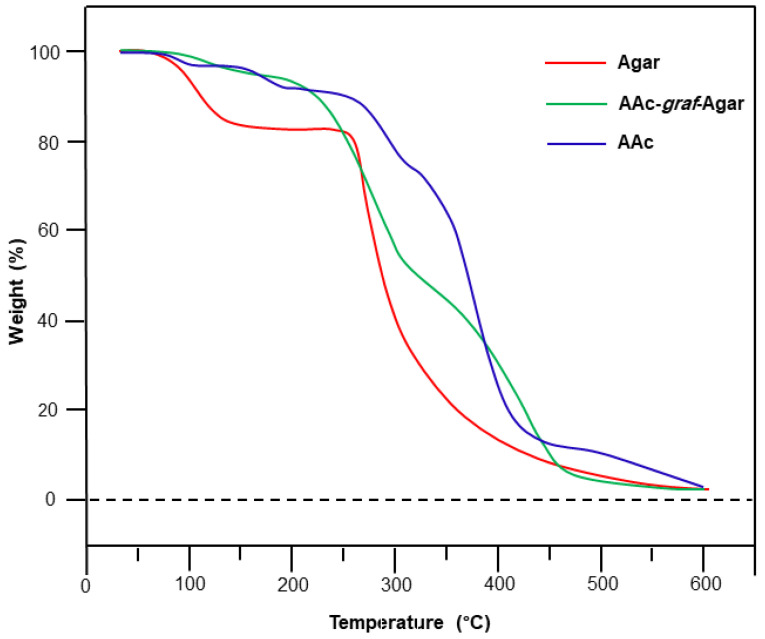
TGA thermograms of AAc-*graf*-Agar hydrogel and its pure components.

**Figure 4:**
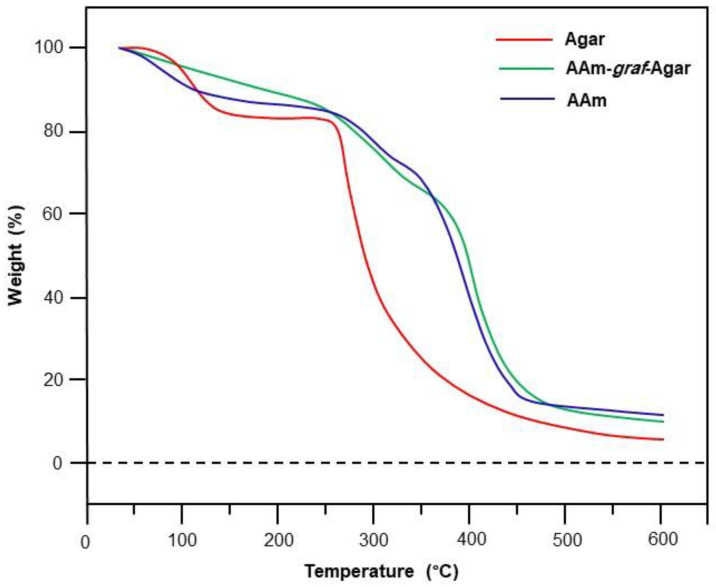
TGA thermograms of AAm-*graf*-Agar hydrogel and its pure components.

The thermal stability of the prepared AAc-*graf*-Agar and AAm-*graf*-Agar hydrogels was investigated by the TGA, and their thermograms obtained are grouped in Figure 3 and Figure 4. As can be observed from the thermal curve of the pure Agar, the decomposition of this polymer takes place in a single step which begins at about 250 °C, which agrees with the literature [27]. The mass loss observed before this temperature (50–150 °C) is probably due to the vaporisation of water molecules incrusted between the chains of this polymer. During this step, about 18 wt% of the water has been volatilised. The profile of the thermal curve of the native PAAc reveals in this figure four stages of decomposition of this material without counting that which characterises the volatilisation of 2.3 wt% of the free water molecules (moisture) observed between 80 and 100 °C which agrees that reported by Moharram and Khafagi [34,38]. The first stage of the decomposition process starts at about 150 °C. During this period, 2.8 wt% of the total mass is released. The second stage begins at 250 °C, in which about 27 wt% of this polyelectrolyte is lost. In this region, a decarboxylation process occurred during this period. The third stage starts at 320 °C, during which 42 wt% of this polymer is released, resulting from a free radical reaction leading to the formation of anhydride by releasing water molecules [38]. Finally, the last step starts at 440 °C. During this period, 13 wt% of this polymer is volatilised, resulting from the main chain scission releasing molecules of ethylene, propylene and saturated and unsaturated rings. On the other hand, the thermogram profile of the AAc-*graf*-Agar copolymer shows the evaporation of free water molecules (moisture) at 100 °C as in the case of pure PAAc; in addition, two main decomposition steps are observed. The process of the first one begins at 200 °C, which is localised between those of the first two stages of the decomposition of the two pure homopolymers and the second at 305 °C which coincides with that of the third step of that of the pure PAAc. This finding indicates a significant increase in the thermal stability of both PAAc and Agar. On the other hand, the thermogram of PAAm homopolymer shows in Figure 4 two main zones of decomposition, the first of which begins at 257 °C in which 15 wt% of the total mass of this polymer is lost, and the second zone of the decomposition process begins at 340 °C. A volatilisation of about 15 wt% of free water molecules embedded in this hydrophilic polymer is observed while the decomposition process begins and ends at approximately 125 °C. Concerning the AAm-*graf*-Agar hydrogel, the profile of its thermogram indicates three main stages of decomposition of this copolymer. The first stage begins at about 30 °C and ends at 250 °C, in which a loss of 18% by weight of the mass of the starting sample is observed. This weight loss regroups free water molecules embedded between the polymer chains and results from dehydration reactions which begin at approximately 150 °C. The second step starts at 360 °C and lasts until the end of the decomposition process, which generates monomer, dimer, CO_2_ and other small molecules resulting from the fragmentation of the copolymer chain backbone, thus showing stability comparable to that of poly(acrylic acid).

#### 2.1.3. SEM Analysis

**Figure 5:**
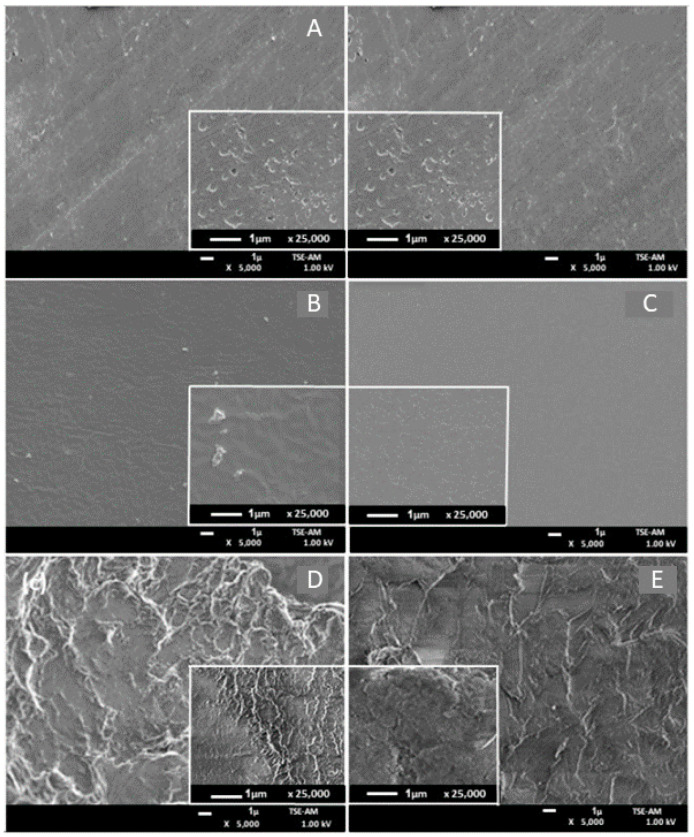
SEM micrographs of Film samples of: (**A**) Agar-Agar; (**B**) Polyacrylamide; (**C**) AAm-*graf*-Agar; (**D**) Poly(acrylic acid), and (**E**) AAc-*graf*-Agar.

SEM images of AAm-*graf*-Agar and AAc-*graf*-Agar copolymer hydrogels and their corresponding homopolymers are shown in Figure 5. The photo of the Agar film sample duplicated to complete in the square offers a rough and porous surface whose pore diameter is ranged between 100 and 500 nm; similar images were also obtained for this biopolymer in the literature [39]. The idea of the polyacrylamide film sample (B), as well as that of the poly(acrylic acid) (C), shows a smooth and slightly wavy surface morphology which is a little less visible in the case of the acidic polymer. The wavy surface seen in these photos is likely formed during the vacuum drying. The difference between the surface morphology of the two synthetic polymers and that of the Agar-Agar dried under the same conditions seems to be mainly due to the nature and the micro-structure of the latter.

Concerning the AAm-*graf*-Agar, the micrograph of this copolymer hydrogel (D) shows a very rough surface that looks like waves of seawater ending up on the edges of a beach. The wrinkling bounded by the white areas observed on the surface of this material in this photo is probably caused by intense, attractive forces due to the intra- and inter-hydrogen bond rearrangement between the hydroxyl groups of the Agar and the amide group of polyacrylamides. The grafting also induced a significant change in the structure of the Agar biopolymer. Thus, it is clear that following grafting, the flaky morphology of the Agar is lost and replaced by a fibrillar and porous morphology. On the other hand, the AAc-*graf*-Agar hydrogel (E) shows rough terrain resembling damage left on the ground after a flood of mud produced by torrential rain.

### 2.2. Swelling Behaviour of Hydrogls

The swelling kinetics of polymer hydrogels can be divided into controlled swelling following Fickian diffusion and relaxation-controlled diffusion following non-Fickian diffusion. The swelling kinetics of a given hydrogel in water is controlled by the diffusion phenomenon when the absorbed molecules diffuse into the polymer matrix much faster than the relaxation of the polymeric chains [40].

**Figure 6:**
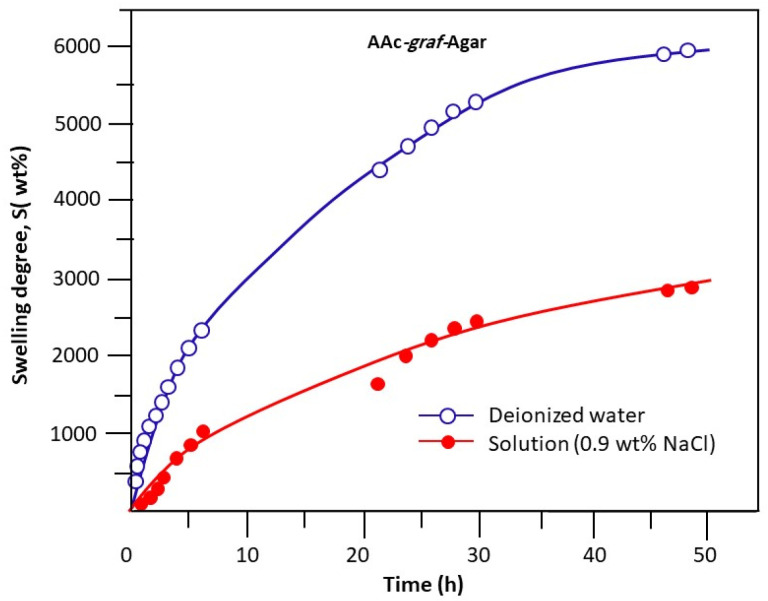
Variation of the swelling degree vs. time of AAc-*graf*-Agar hydrogel in deionised water and saline solution containing 0.9 wt% of at 25 °C.

**Figure 7:**
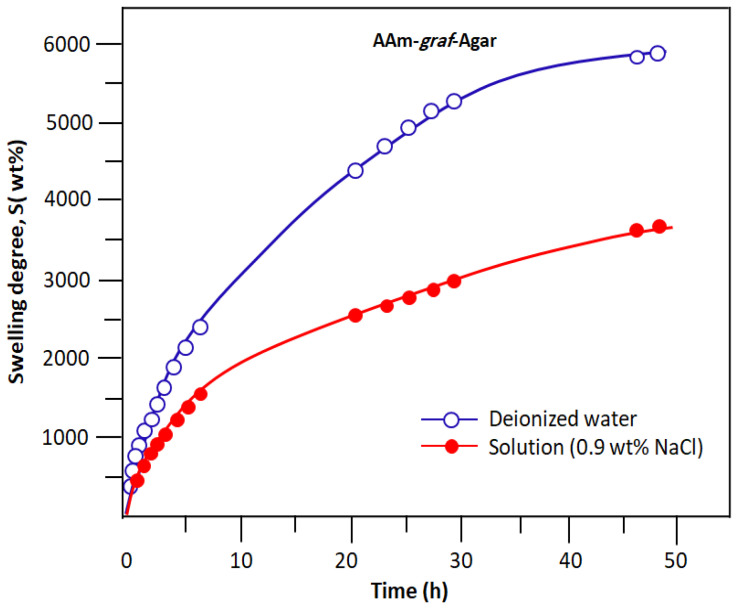
Variation of the swelling degree vs. time of AAm-*graf*-Agar hydrogel in deionised water and saline solution containing 0.9 wt% of at 25 °C.

In this work, the swelling degree of AAc-*graf*-Agar and AAm-*graf*-Agar hydrogels versus time was studied during 48 h at 25 °C in deionised water and salted water containing 0.9 wt% NaCl and the results obtained are plotted in Figure 6 and Figure 7, respectively. Usual pseudo-logarithmic curves are obtained, and the swellability, which corresponds to the maximum swelling or the swelling equilibrium for the AAc-*graf*-Agar hydrogel, reached 6000 wt% in deionised water and 2800 in salted water. On the other hand, the swellability of the AAm-*graf*-Agar hydrogel was found to be 5700 and 3600 wt% in deionised and salted water, respectively.

As can be observed from the profile of these curves, Agar grafted with poly(acrylic acid) exhibits higher swelling capacity than grafted with polyacrylamide. This can be attributed to the presence of -COO groups along the macromolecular chains of poly(acrylic acid) and also to the increase in the number of free H^+^ ions (counter ions) in the gel phase. Hence, this leads to an increase in chain relaxation due to repulsion between -COO groups of similar charge [41]. Acrylic polymers can create hydrogen bonds and stiff frameworks in aqueous solutions, which explains why these suspensions typically take on a gel-like appearance. Several steps are involved in how acrylic polymers interact with water. Prior to the macromolecules relaxing and forming a polymer solution, water first diffuses heavily into the polymer bulk and causes active hydration of the macromolecules [42].

Due to ex-osmosis, hydrogels do not swell much in the presence of electrolyte salts, and even swollen hydrogels notably shrink in the presence of salts [43]. This phenomenon is due to the action of the Na^+^ counter-ion, which leads to the collapse of the internal network of the polymer. Since the concentration of counter-ions (in this case, Na^+^ ions) is so high in the solution, they condense around the fixed -COO charges, decreasing the repulsive forces between the -COO groups along the polymer segments and inducing a reduction in the degree of swelling. The increase in water salinity leads to the increase of the osmotic pressure that occurs from the less concentrated medium (hydrogel) to the more concentrated medium (salted water), reducing the percentage of swelling [44].

### 2.3. Adsorption Parameters

#### 2.3.1. Point of Zero Charge (pH_pzc_)

**Figure 8:**
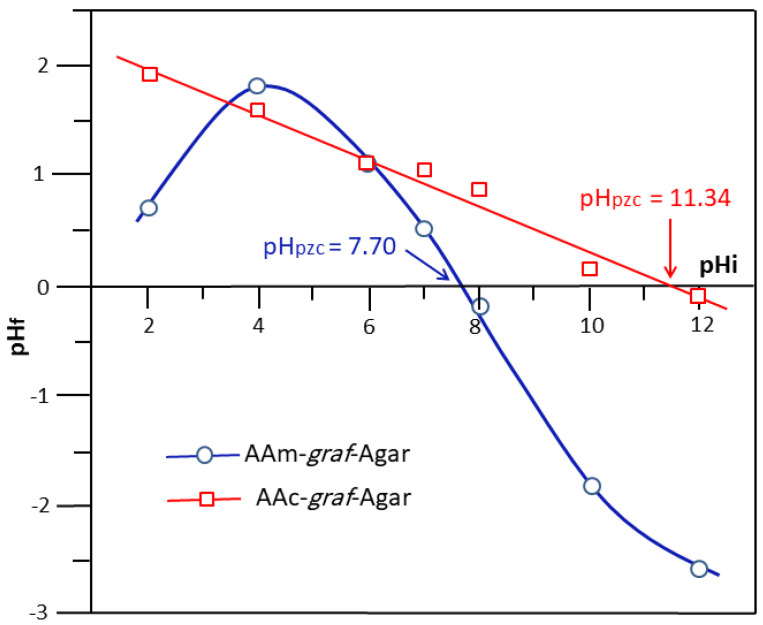
Adsorption parameters, point of zero charges of PAAm-*graf*-Agar and PAAc-*graf*-Agar.

pH_pzo_ is a crucial parameter used to determine the surface charge character of a hydrogel. In this work, the determination of this parameter was deduced from the curve indicating the variation of the final pH versus the initial pH of the solution, as shown in Figure 8. As noted in this figure, the values of pH_pzc_ for PAAm-*graf*-Agar and AcA-*graf*-Agar hydrogels are 7.7 and 11.34, respectively. According to Harrach et al. [45], for pH values higher than pH_pzc_, the negative charge of the surface of the hydrogels facilitates the adsorption of cationic molecules. On the other hand, for pH values below pH_pzc_, the positive charge of the activated carbon surface is able to repel cations [45], which agrees with the results obtained.

#### 2.3.2. Effect of Initial pH Medium

**Figure 9:**
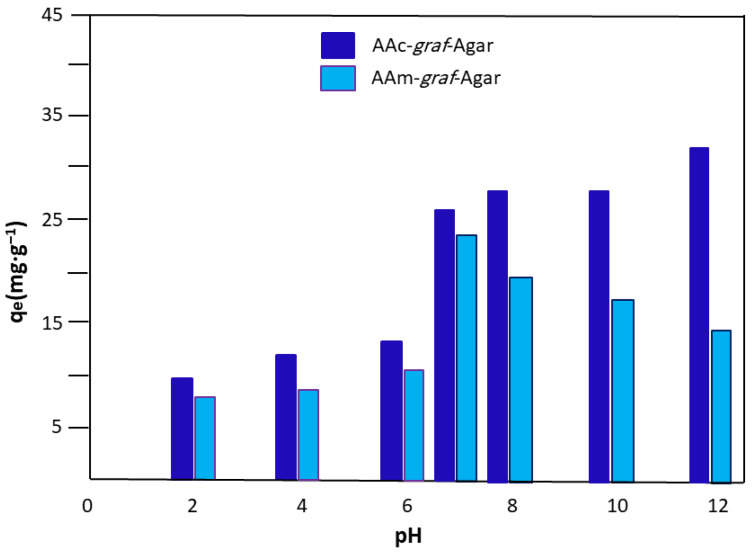
Variation of the adsorption capacity versus the pH medium.

The initial pH of the adsorbed medium is a key factor in the adsorption process, as this In parameter can influence the surface ionisation and the charge of the adsorbent [42,46]. In this work, the change in the adsorption capacity, q_e_ (mg∙g^−1^), versus the pH of medium for the PAAc-*graf*-Agar and PAAm-*graf*-Agar hydrogels are gathered for comparison in Figure 9.

First, in all measured pH ranges, PAAc-g-Ag hydrogel had better adsorption efficiency than PAAm-g-Ag hydrogel. Second, the adsorption effectiveness was dramatically decreased for pH values as low as 4 or as high as 9, respectively. As can be seen from the histograms obtained, the PAAc-*graf*-Agar hydrogel has better adsorption efficiency compared to that of PAAm-*graf*-Agar in all the measured pH media ranges. These results also reveal that, for the PAAc-*graf*-Agar, the adsorption efficiency increased with the pH medium, while for the PAAc-*graf*-Agar, the *q_e_* passed by a maximum in a neutral pH medium. At pH = 7.7 for the PAAm-*graf*-Agar hydrogel and at pH = 11.34 for the PAAc-*graf*-Agar hydrogel, the highest adsorption rates are achieved, as shown in this figure. In this situation, electrostatic interactions between the molecules of the dye and the surface of the hydrogels, generated by the H^+^ ions in an acid medium and the OH^-^ ions in an alkaline medium, limit the effectiveness of the adsorption [47,48].

#### 2.3.3. Effect of Initial Concentration of Dye

**Figure 10:**
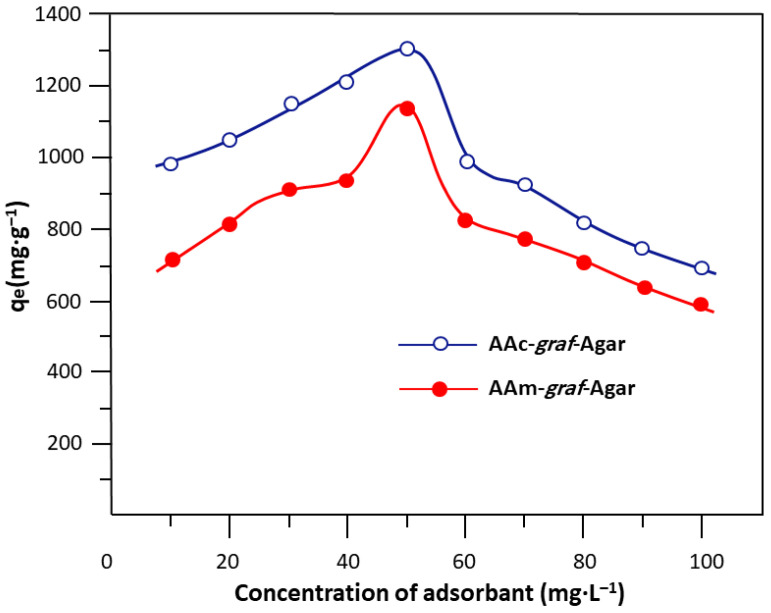
Variation of the adsorption capacity, q_e_ (mg∙g^−1^), vs. the initial dye concentration.

One of the key elements in the adsorption process is the initial concentration of pollutants since it is thought to act as a catalyst for overcoming the mass transfer resistance between solid (adsorbent) phases and aqueous solutions [49]. For the two prepared hydrogels, the impact of the initial concentration of pollutants (dyes) on the adsorption efficiency is illustrated in Figure 10. As can be observed through the profile of these curves, the adsorption efficiency for both types of adsorbents evolves according to the same trend, which increases linearly with the concentration of the dyes until reaching a maximum of 1032 mg∙g^−1^ (AAc-*graf*-Agar) and 1000 mg∙g^−1^ (AAm-*graf*-Agar) for a dye content of 1250 mg∙L^−1^ then stabilises or slightly decreases beyond [50].

#### 2.3.4. Effect of Adsorbent Dose

Another important factor influencing the adsorption efficiency of pollutants and, more particularly, dyes is the adsorbent content [51]. In this study, the adsorption capacity of the PAAc-*graf*-Agar and PAAm-*graf*-Agar hydrogels is evaluated under the following conditions: starting dye concentration of 50 mg·L^−1^, initial pH of the absorbent medium of 12 (PAAc-*graf*-Agar hydrogel) and 7 (PAAm-*graf*-Agar) and the contact period of 24 h.

**Figure 11:**
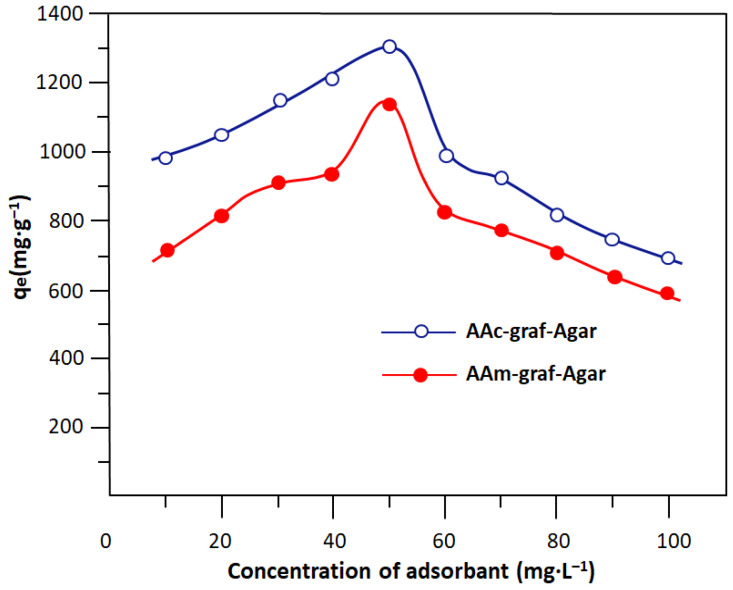
Variation of the adsorption capacity, q_e_ (mg∙g^−1^), vs. the dose of adsorbent.

The evolution of the adsorption capacity of the AAc-*graf*-Agar and AAm-*graf*-Agar hydrogels as a function of the concentration of the adsorbent in the absorbate medium is plotted in Figure 11. The profiles of these curves obtained have practically the same trend for the two hydrogels studied and show that the variation of *qe* versus the concentration of the absorbent passed through a maximum of 1300 mg·g^−1^ (AAc-*graf*-Agar) and 1130 mg·g^−1^ (AAm-*graf*-Agar) when the absorbent content was 50 mg·L^−1^. The efficiency of the dye adsorption is improved for both hydrogels until the dosage in the adsorbent reaches 50 mg·L^−1^. This testifies to the great absorption capacity of these two materials under these conditions, while its rapid decrease at lower doses is probably due to a depletion of the active amount of component which has formed and of the dye in the medium. A similar explanation was reported by Garcia et al. [52] using biomass sorption on Macrocystis pyrifera to remove reactive Black-5. These results also reveal that at all the adsorbent doses examined, the adsorption efficiency of the acrylic acid hydrogel was higher than that of the acrylamide hydrogel because the surfaces of the PAAc-*graf*-Agar contain higher concentrations of functional groups, including -COOH, phenolic -OH, phosphates, and amino acid components of acrylic acid [53].

#### 2.3.5. Effect of Contact Time

**Figure 12:**
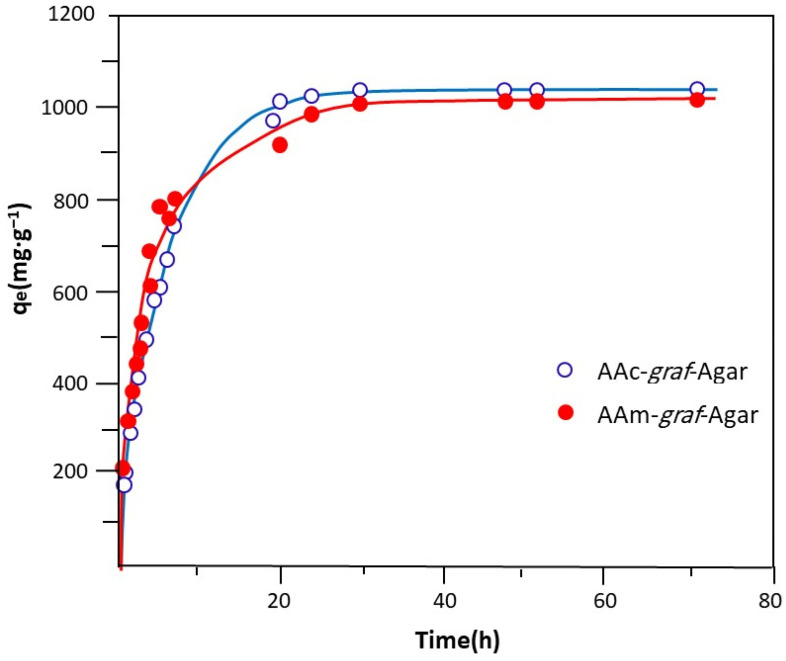
Variation of the adsorption capacity, q_e_ (mg∙g^−1^), vs. time.

Figure 12 illustrates how increasing the contact time increased the amount of MB dye adsorbed. The starting dye concentrations used in the adsorption procedure are 1200, 1400, and 50 mg∙L^−1^ for PAAc-*graf*-Agar, PAAm-*graf*-Agar, and adsorbent, respectively. Each mixture is stirred at a stirring rate of 100 rpm and at room temperature (~25°). The adsorption capacity of these two hydrogels followed almost the same trend increasing logarithmically with a slight increase observed in the case of the hydrogel containing acrylic acid. This increase is faster during the first 10 h (119 mg·g^−1^·h^−1^) for AAc-*graf*-Agar and (146 mg·g^−1^·h^−1^) for AAm-*graf*-Agar, then slowly (2.67 mg·g^−1^ h^−1^) for AAc-*graf*-Agar and (4.0 mg.g^−1^·h^−1^ for AAm-*graf*-Agar to reach equilibrium (saturation) when the q_e_ value was1040 mg·g^−1^ for the first hydrogel and 1010 mg·g^−1^ for the second hydrogel which is obtained at 30 h of the adsorption process [54].

#### 2.3.6. Effect of Temperature

**Figure 13:**
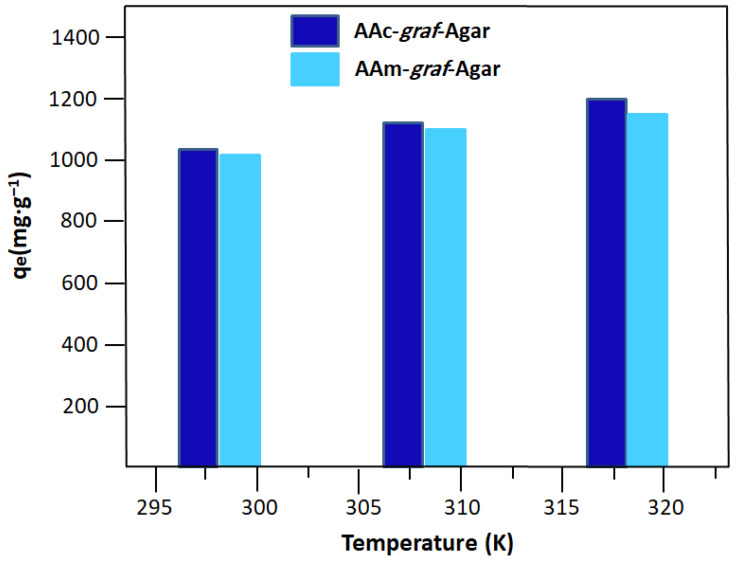
Effect of temperature on the adsorption capacity.

The impact of temperature on the percentage of dye removal was examined at 25, 35 and 45 °C in a thermostatic bath after adjusting the different adsorption parameters, and the data obtained are histogram in Figure 13, as can be seen from these results that the adsorption capacity at the equilibrium of these two hydrogels increased with temperature, which passed from 1040 to 1200 mg·g^−1^ (AAc-*graf*-Agar) and from 1020 to 1160 mg·g^−1^ (AAm-*graf*-Agar). This indicates that the adsorption effect was improved, and the time to reach the steady state was reduced due to the appropriate temperature increase. The swelling properties of the hydrogel composite and the mobility of the dye ions can both be accelerated by higher temperatures which go in the direction of increasing the porosity of the absorbent [54].

**Figure 14:**
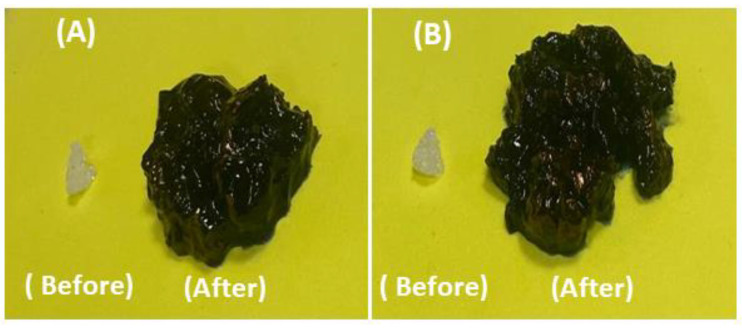
AAm-*graf*-Agar (**A**) and AAm-*graf*-Agar (**B**) photos were taken before and after the MB dye adsorption process.

Figure 14 shows photos of AAc-*graf*-Agar (A) and AAm-*graf*-Agar (B) hydrogel samples taken before and after the adsorption process in the medium containing MB. As can be observed from these images taken at the equilibrium of the adsorption process that, these specimens swelled in the MB solutions in which the q_e_ were 1032 and 1000 mg·g^−1^ for the hydrogels containing acrylic acid and acrylamide, respectively.

### 2.4. Adsorption Isotherm Models

The determination of the maximum adsorption capacity of the MB dye on the absorbent is crucial. In this work, to reach this goal, models of adsorption isotherms as formulated by Langmuir (Equation (1)) [55], Freundlich (Equation (2)) [56] and Temkin (Equation (3)) [57] were applied to evaluate this parameter in the AAc-*graf*-Agar and AAm-*graf*-Agar hydrogels used as adsorbents.

(1) $$q_{e}=\frac{q_{m}\times K_{L}\times C_{e}}{1+K_{L}\times C_{e}},\;\mathrm{with}\;R_{L}=\frac{1}{1+K_{L}+C_{e}}$$

where *q_m_* and *K_L_* are the maximum adsorption capacity of dye (mg·g^−1^) and the Langmuir constant, respectively. *R_L_* and $C_{e}$ are the separation factor and the equilibrium concentration of dye, respectively. Noting that the $R_{L}$ value defines the isotherm as linear ($R_{L}$ = 1), unfavourable ($R_{L}$ > 1), and favourable (0 < $R_{L}$ < 1).

**Figure 15:**
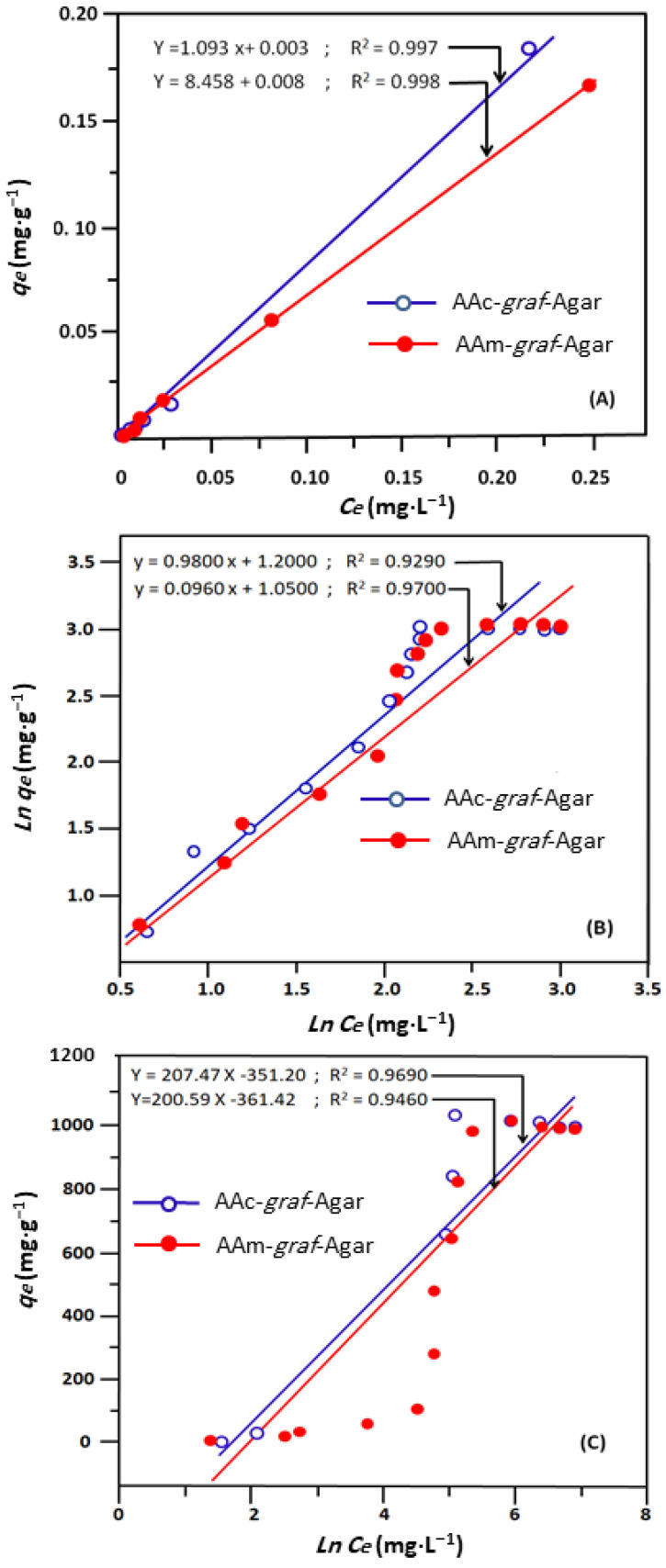
Linear relationship of (**A**) Langmuir; (**B**) Freundlich and (**C**) Temkin isotherm models for the adsorption of MB dye from aqueous solution using PAAc-*graf*-Agar and PAAm-*graf*-Agar hydrogels.

(2) qe=KfCe1n

where $q_{e}$ is defined earlier, $K_{f}$ is the distribution coefficient, and *n* is a correction factor

(3) $$q_{e}=B\ln(A\times C_{e}),\;\mathrm{with}\;B_{T}=\frac{RT}{K_{T}}$$

where *A* (g^−1^) and *B_T_* (kJ/mol) are Temkin constants, respectively. *R* and *T* are the gas constant and the absolute temperature (*K*), respectively. Figure 15 shows the isothermal curves of MB adsorptions on the surfaces of the two prepared hydrogels by applying the Langmuir, Freundlich and Temkin models. Table 1 shows the comparison of the adsorption parameters determined using Equations (1)–(3) and the correlation coefficients which accompany them. The comparison of the shapes of the curves obtained reveals that the Langmuir model applied to the adsorption of the MB dye on the prepared hydrogels is the best suited to the concentration range investigated. Because this model gives better correlation coefficients for these two MB-hydrogel systems (*R*^2^ = 0.9970 for PAAc-*graf*-Agar and (*R*^2^ = 0.998 for PAAm-*graf*-Agar hydrogel).

According to the values of the *B_T_* and *K_T_* parameters of the Temkin isotherm, there is only a weak interaction between the MB dye and the surface of the adsorbent, which is consistent with the results for other categories of hydrogels [58].

The *R_L_* values for AAc-*graf*-Agar and AAm-*graf*-Agar hydrogels, which are 0.84 and 0.94, respectively, indicate that the adsorption of MB by these two hydrogel systems is a very advantageous method. Moreover, the low *R_L_* value for AAc-*graf*-Agar reveals that the grafting of the acrylic acid units on the Agar chains increases the adsorption amounts of MB. A similar result was also obtained by Zhu et al. [59] on the adsorption of this dye on biochar surfaces.

The q_m_ values of AAc-*graf*-Agar and AAm-*graf*-Agar hydrogels relative to MB dye are 1035.6 and 1015.96 mg·g^−1^, respectively. This indicates that the incorporation of acrylic acid units into the Agar chains improves the absorbability of this biopolymer hydrogel, as can also be seen from these data that the maximum adsorption capacity of AAc-*graf*-Agar is within the MB adsorption range examined by other researchers on other types of hydrogels [60].

The application of the Freundlich and Temkin models on the adsorption of the MB dye on the two prepared hydrogels both led to pseudo-straight lines characterised by correlation factors, *R*^2^, greater than 0.95. According to Nath et al. [5], the application of the equations generated from these two models can also be used to approximate the multilayer adsorption behaviour of MB dye on AAc-*graf*-Agar and AAm-*graf*-Agar hydrogels which is advantageous.

The heterogeneity factor is also another parameter which makes it possible to describe the adsorption process. The adsorption capacity, *K_F_*, gives an idea of the strength of the adsorption bond. The *K_F_* value for adsorption onto AAc-*graf*-Agar (2.26) is higher than that on AAm-*graf*-Agar (1.93). This indicates that the layer of copolymer based on acrylic acid and Agar improves the adsorption capacity of the hydrogel [58].

### 2.5. Adsorption Kinetics

The study of the adsorption kinetic was conducted using the experimental conditions of Table 2. To assess the adsorption kinetics process of MB dye onto the AAc-*graf*-Agar and AAm-*graf*-Agar hydrogels, two kinetic models, the pseudo-first-order (Equation (4)) [53,57] and pseudo-second-order (Equation (5)) [58] were considered.

The following equation gives the kinetic model of the pseudo-first-order:

(4) $$\frac{dQ}{dt}=k_{1}(Q_{e}-Q_{t}),$$

Integrating of Equation (4) yields the equation in a linear form:

(5) $$\ln\left(Q_{e}-Q_{t}\right)=lnQ_{e}-k_{o}t,$$

The following relationship is used to represent the kinetic model of the pseudo-second-order:

(6) $$\frac{dQ}{dt}={k_{2}(Q_{e}-Q_{t})}^{2},$$

Equation (6) can be represented in its linear form as follows:

(7) $$\frac{1}{Q_{t}}=\frac{1}{k_{2}Q_{e}^{2}}+\frac{1}{Q_{e}}t,$$

The kinetic constants and parameters obtained for the adsorption process of MB dye onto the surfaces of the prepared hydrogels are gathered for comparison in Table 3.

**Figure 16:**
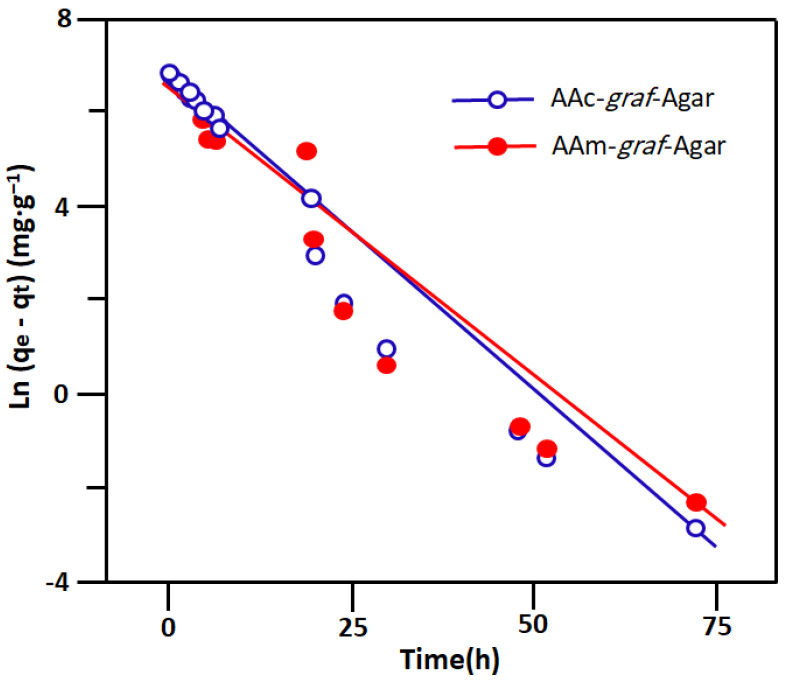
Kinetic plots for MB dye adsorptions of pseudo-first order on AAc-*graf*-Agar and AAm-*graf*-Agar hydrogels samples.

**Figure 17:**
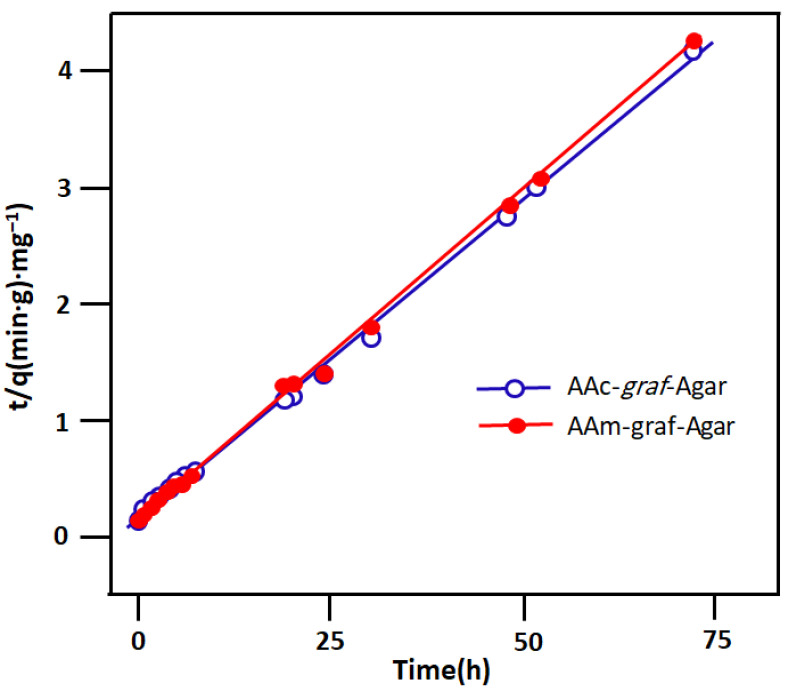
Kinetic plots for MB dye adsorptions of the pseudo-second-order model on AAc-*graf*-Agar and AAm-*graf*-Agar hydrogels samples.

The kinetic models of pseudo-first-order and pseudo-second-order were built in accordance with the results of adsorption experiments, as shown in Figure 16 and Figure 17, respectively. Table 3 provides a summary of the statistical parameters for various adsorptions. The correlation coefficients were used to evaluate which of the adsorption kinetic models suited the data the best (*R*^2^). It was revealed that the *R*^2^ value for the pseudo-second-order model was higher (0.999) and nearly equal to that of the other model (0.933).

This table presents a summary of the statistical parameters for various adsorptions. Based on the *R*^2^ values to know which of the kinetic models of adsorption best suited the data obtained. It was found that the value of the correlation coefficient of the pseudo-second-order model is well higher (0.999).

The theoretical calculation gave a maximum adsorption capacity of 1035.96 mg·g^−1^ for the AAc-*graf*-Agar hydrogel, which is relatively similar to that experimentally obtained (q_e_ = 1030 mg·g^−1^). From the profiles of the previous curves and the *R*^2^ values, it was demonstrated that the adsorption of the MB dye on the surfaces of the two hydrogels obeyed the second-order kinetic model. This suggests that the availability of reactive sites on the surface of hydrogels affects the adsorption of the MB dye [61]. This also indicates that the adsorption phenomenon involved in this study follows a chemical process. Table 4 shows a comparison between the values of q_max_ obtained in this work with those of the literature.

The comparison of the results obtained in this work with those of the literature shows that the adsorption capacity of the hydrogels AAc-*graf*-Agar and AAm-*graf*-Agar is clearly better than some obtained by other adsorbents published in the literature. In other words, the greatest contribution of this study was not only dedicated to improving the process of removal of organic dyes like MB from wastewater but also its application in the biomedical industry.

### 2.6. Recovery, Regeneration, and Reusability

For a hydrogel to be reusable as an adsorbent, it must meet at least two of the most critical requirements, which are reversible adsorption and stability. Stability refers to the resistance of the hydrogel to typical environmental conditions. The hydrogel must be chemically, biologically and physically stable during consecutive adsorption–desorption cycles. In this work, the synthesised hydrogels are cross-linked hydrophilic polymer networks with high water content (3000–5980 wt%). Due to the weak mechanical properties and the fragile structure of the prepared hydrogels [66] due to the presence of the Agar-Agar units [67], after a certain period of reuse, the excessive swelling causes a dislocation of the polymer network (fragmentation of the hydrogel) testifying to its low mechanical resistance to the pressure exerted by water molecules on the bonds of the agar-agar R-O-R type. Therefore, the recovery and reuse of these hydrogels as an absorbent of MB dye are not always easy to achieve, and it depends on several factors, including the nature of the solvent used [3]. Nevertheless, its biocompatibility and flexibility [39,66,67,68,69,70] makes it possible to recycle the absorbent/absorbate assembly in several fields of industry involving water-soluble dyes. Indeed, the desorption process of MB dye from AAc-*graf*-Agar/MB and AAm-*graf*-Agar/MB hydrogels was carried out in ethanol containing 0.1 M hydrochloric acid in which 98 and 85 wt% of this dye has been recovered, respectively after the first cycle of use. On the other hand, the MB dye desorption in deionised water was very low for the two hydrogels examined. Such results were also observed by Duman et al. [3] uses as absorbent a composite-based hydrogel involving agar-agar and κ-carrageenan. In the first recycling period, 63.12 and 45.83 wt% of this dye were desorbed in pure ethanol for the hydrogel based on acrylic acid and 45.83 wt% for that containing acrylamide. A slight depression in the capacity of desorption was noted for the two hydrogels in the 5th period of the MB sorption–desorption process.

## 3. Conclusions

The goal of this investigation has been achieved. Indeed, hydrogels involving acrylic acid and Agar-Agar (AAc-*graf*-Agar) and acrylamide and Agar-Agar (AAm-*graf*-Agar) were successfully synthesised by grafting acrylic or acrylamide units on the Agar-Agar biopolymer by free radical polymerisation route. The thermal degradation of AAc-*graf*-Agar and AAm-*graf*-Agar hydrogels indicated starting decomposition temperatures at 200 and 250 °C, respectively, indicating a significant increase in stability of AAm-*graf*-Agar comparable to that of AAm-*graf*-Agar. The application of the prepared hydrogel as an adsorbent of methylene blue dye has given very satisfactory results. Indeed, the adsorption efficiency of the MB dye revealed better adsorption efficiency for AAc-*graf*-Agar than AAm-*graf*-Agar hydrogels, and the adsorption effectiveness decreased depending on the pH media. For both hydrogels, the adsorption efficiency increased with the pH medium and the highest adsorption rates was achieved in pH media 7.7 and 11.34 for the AAm-*graf*-Agar and AAc-*graf*-Agar hydrogels, respectively. For the two prepared hydrogels, the efficiency of the dye adsorption was improved until the dosage in the adsorbent reached 50 mg·L^−1^ and increased with the contact time and the temperature. It was found, from the kinetics data, that the Langmuir model applied to the adsorption of the MB dye on the prepared hydrogels was the best suited in the concentration range investigated and was best fitted by the pseudo-second-order kinetic model. The values of BT and KT of the Temkin isotherm obtained indicated weak interactions between the MB dye and the adsorbent surface, while the RL values of these hydrogels (0.84–0.94) indicated that the adsorption of MB by these materials is very advantageous as a method to remove dye from water. The KF value for adsorption onto AAc-*graf*-Agar (2.26) is higher than that on AAm-*graf*-Agar (1.93), indicating that the layer of copolymer based on acrylic acid and Agar improves the adsorption capacity of the hydrogel. The recovery, regeneration, and reusability tests of these prepared hydrogels in pure ethanol and ethanol containing 0.1 M HCl revealed slight depression in the capacity of desorption for the two hydrogels in the 5th period of the MB sorption–desorption process and an excessive swelling causes dislocation and fragmentation of the polymer network.

## 4. Materials and Methods

### 4.1. Materials

Agar-agar (Agar) (98%) was provided by Scharlau (Barcelona, Spain), Acrylic acid (AAc) (99%) and Acrylamide (AAm) (98%) were purchased from Sigma-Aldrich (Darmstadt, Germany) and Biochem (Cosne-Cours-sur-Loire, France), respectively. Acrylamide was purified by recrystallisation in methanol and dried in a vacuum desiccator; acrylic acid was purified from hydroquinone by distillation under a vacuum.

Ammonium peroxodisulfate (APS) (98%) and methylene blue (MB) (96%) were supplied by Panreac Monplet & Esteban (Barcelona, Spain) and Biochem (London, ON, Canada), respectively. Ethanol (95.6%) was obtained from Biochem Chemopharma (Cosne-Cours-sur-Loire, France). All of these reagents and solvents were used without prior purification.

### 4.2. Preparation of PAAm-graf-Ag and PAAc-graf-Ag Hydrogels

A solution containing 1.0 g of Agar dissolved in 40 mL of deionised water and another solution containing a known amount of acrylamide or acrylic acid dissolved in 10 mL of deionised water were mixed together to form a ternary solution grouping the Agar, acrylamide or acrylic acid and water. 0.007 g of ammonium peroxodisulfate, used as an initiator, was then added to the reaction mixture. The whole was kept under bubbling of nitrogen gas and with moderate stirring for 35 min. The reaction mixture was then placed in a glass tube with an inside diameter of 10 mm and left for 24 h at room temperature (~25°). The reaction mixture is placed in a glass tube with an internal diameter of 10 mm and left at ambient temperature (~25 °C) for 24 h. When the polymerisation and crosslinking processes are completed, the hydrogel is then removed from the tube and then cut into square pieces of 1 cm side. To purify the prepared material from the residual reactants, the hydrogel was washed several times with deionised water, then with 80% ethanol/water mixture and then allowed to dry at room temperature for 48 h and under vacuum at 45 °C to constant weight. The preparation conditions of AAc-*graf*-Agar and AAm-*graf*-Agar hydrogels are grouped in Table 5, and the percentage of grafting, *G* (%), was calculated using the following equation [71]:

(8) $$G\left(\%\right)=\frac{{Wt}_{p}-{Wt}_{ps}}{m_{ps}}\times 100,$$

where ${Wt}_{p}$ and ${Wt}_{ps}$ are the weights of graft polymer and polysaccharide, respectively.

### 4.3. Characterisation

The FTIR spectra of Agar, AAc-*graf*-Agar and AAm-*graf*-Agar hydrogels were performed on a NICOLET10-Thermoscientific Model FT-IR Spectrophotometer. Thin film samples of the dried hydrogels were scanned in the wavenumber range from 400 to 4000 cm^−1^.

The TGA thermograms of the polymer samples were plotted on a TG/DTA 12 instrument (SETARAM Labsys^TM^). Dried samples were placed in aluminium pans and introduced in the TGA cell, then heated from 20 to 600 °C with a heating rate of 20 °C∙min^−1^ under an airflow.

The micrographs of the dry film samples were taken by a scanning electron microscope JEOL, JSM7610FPlus (Tokyo, Japan) at various magnifications after having been coated with the gold grid. These micrographs were taken at an accelerating voltage of 17 kV.

### 4.4. Swelling Behaviour

In this work, the gravimetric approach was used to measure the swelling rate of prepared hydrogels in aqueous media. To achieve this goal, dry hydrogel samples were first weighed on a precision balance and then immediately immersed in excess deionised water maintained at 25 °C for 48 h. These specimens were then removed from the medium at known intervals of time, gently wiped on both sides with absorbent paper in order to eliminate the droplets of water deposited on the surfaces then immediately weighed. This operation continued until the saturation of the samples, which is characterised by a constant weight. This experiment was repeated under various environmental conditions, and the equilibrium percentage swelling (swelling at saturation) of hydrogels is calculated using Equation (9) [72]

(9) $$S\left(wt\%\right)=\frac{w_{t}-w_{o}}{w_{o}}\times 100,$$

where $w_{o}$ and $w_{t}$ are the masses of the dried and swollen hydrogel at a time *t* of the swelling process.

### 4.5. Adsorption Experiment

For the adsorption examinations, each film sample of dry hydrogel was immersed in an aqueous solution containing methylene blue at a concentration varying between 10 and 2000 mg·L^−1^. During this experiment, the samples are shaken at 100 rpm in an incubator for 24 h at 25, 35 and 45 °C. This experiment was also carried out on the effect of the pH of the medium. To achieve this goal, aqueous solutions of pH 2, 4, 6, 7, 8, 10 and 12 were prepared by adjusting the pH value by adding an adequate amount of hydrochloric acid for acidic media and NaOH for basic media. UV-Vis spectroscopy was used to calculate the residual concentration of MB in the solutions after the adsorption process using a calibration curve indicating the variation of the concentration vs. the absorbance. The values of the adsorption capacity, *q_e_* (mg·g^−1^), were determined using Equation (10):

(10) $$q_{e}=\frac{\left(C_{0}-C_{e}\right)*V}{m},$$

where *V* (L) is the sample solution volume, and *m* (g) is the mass of the hydrogel. *C*_0_ and *C_e_* (mg·L^−1^) are the starting and interval concentrations of MB, respectively.

### 4.6. Determination of the Point of the Zero Charge (pH_pzc_)

In the adsorption process, the pH of the medium is an essential parameter which affects the adsorption phenomenon. Its effect on the hydrogel adsorption is governed by the pH_pzc_ value of the samples. Indeed, when the pH medium is lower than the value of pH_pzc_, this indicates that the surface of the hydrogel is positive and that there is an electrostatic repulsion between the surface of the positively charged hydrogel and the MB, and consequently, this leads to lower sorption. When the pH of the solution is higher than the pH_pzc_ value, this indicates that the hydrogel surface is negatively charged, and therefore, this leads to more attraction between the MB ions and the hydrogel surface. This promotes the increase of MB adsorption capacity.

In this study, the effect of the pH medium on the prepared AAc-*graf*-Agar and AAm-*graf*-Agar hydrogels is realised in a pH medium ranging between 2 and 14.

The point of zero charges (pH_pzc_) was carried out to determine the pH value for which the net surface charges of the hydrogels are zero; For the determination of pH_pzc_, 20 mL of a 0.01 M NaCl solution was kept in a capped 100 mL conical flask, the pH was then modified to a consecutive integer from 2 to 12, by carefully adding the required amount of either 0.1 M HCl or 0.1 M NaOH. The hydrogels (0.05 g) were added to the solution in a conical flask, capped and placed in the shaker. The final pH of all samples was measured at a stirring rate of 100 rpm for 24 h at 300.15 ± 2.00 K. The pH of the solution was measured using a digital pH meter, and the pH_pzc_ was noted when the initial pH of the medium was equal to that of the equilibrium [73,74].

## Figures and Tables

**Table 1:** The parameters of the adsorption isotherms of Langmuir, Freundlish and Temkin for the methylene blue dye onto the prepared Hydrogels.

Hydrogel	Adsorption Isotherm Parameters									
	Langmuir				Freundlich			Temkin		
	*q_max_*	*K_L_*	*R_L_*	*R* ^2^	*n*	*K_f_*	*R* ^2^	*B_t_*	*K_t_*	*R* ^2^
AAc-*graf*-Agar	304.87	0.003	0.84	0.997	1.025	2.26	0.929	207.47	0.184	0.969
AAm-*graf*-Agar	118.22	0.001	0.94	0.998	1.032	1.93	0.970	200.59	0.165	0.901

**Table 2:** Preparation conditions of the adsorption kinetics of MB dye on the surfaces of AAc-*graf*-Agar and AAm-*graf*-Agar hydrogels.

Hydrogel	Initial MB Dye (mg·L^−1^)	Adsorption Dose (mg·L^−1^)	Stirring Rate (rpm)	Contact Period (h)	Temperature (°C)
AAc-*graf*-Agar	1400	50	100	48	25
AAm-*graf*-Agar	1200	50	100	48	25

**Table 3:** Kinetic constants and parameters for the pseudo-first and second order models.

Hydrogel			Pseudo-First-Order Model			
	Ln(q_e_)	*K*_1_ *t*	q_e_ (mg·g^−1^)	*K*_1_	*R* ^2^	1/q^e^ (g·mg^−1^)
AAc-*graf*-Agar	6.7362 ± 0.0238	0.00222 ± 1.89359 × 10^−5^	842.353	5.138 × 10^−7^	0.999	9.67 × 10^−4^ ± 1.0213 × 10^−5^
AAm-*graf*-Agar	6.5217 ± 0.05913	0.00205 ± 4.66628 × 10^−5^	679.746	4.745 × 10^−7^	0.994	9.89 × 10^−4^ ± 7.8794 × 10^−6^
	Pseudo-second-order model					
Hydrogel	q_e_ (mg·g^−1^)	*K*_2_	*R* ^2^		1/k_2_qe^2^ (g^2^·mg^−2^)	
AAc-*graf*-Agar	1030	4.94 × 10^−6^	0.997		0.18922 ± 001621	
AAm-*graf*-Agar	1010	6.55 × 10^−6^	0.998		0.14939 ± 0.01251	

**Table 4:** Comparison of the current values of the q_max_ with those provided in the literature.

Adsorbent	q_max_ (mg·g^−1^)	Adsorbate	Ref.
Agar/κ-carrageenan hydrogel	205.9	MB	[3]
N-isopropylacrylamide/itaconicacid/pumice composite hydrogel	22.18	MB	[62]
Polyacrylamide/polyacrylate/gum Arabic	48.00	MB	[63]
κ-Carrageenan-*graf*-polyacrylamide/bentonite composite hydrogel	156.3	MB	[64]
PTCS/OMMT hydrogel composite	49.01	MB	[54]
Agar/maltodextrin/polyvinyl alcohol-montmorillonite (20%)	71.51	MB	[2]
Poly(AMPS-*co*-IA)	24.1	MB	[65]
AAc-*graf*-Agar	304.84	MB	This work
AAm-*graf*-Agar	118.22	MB	This work

PTCS/OMMT: Purified total corn stalks/Organic montmorillonite; AMPS-*co*-IA: poly(2-acrylamido-2-methylpropane sulfonic acid-co-itaconic acid).

**Table 5:** Preparation conditions of the AAc-*graf*-Agar and AAm-*graf*-Agar hydrogels.

Hydrogel	Agar (g)	AAc (mL)	AAm (g)	APS (g)	G (%)
AAc-*graf*-Agar	1.0	-	10.0	0.007	896
AAm-*graf*-Agar	1.0	10.0	-	0.007	1020

## Data Availability

The data that support the findings of this study are available from the corresponding author upon reasonable request.
